# Outdoor thermal comfort in public space in warm-humid Guayaquil, Ecuador

**DOI:** 10.1007/s00484-017-1329-x

**Published:** 2017-03-10

**Authors:** Erik Johansson, Moohammed Wasim Yahia, Ivette Arroyo, Christer Bengs

**Affiliations:** 1 Present Address: Housing Development and Management, Department of Architecture and Built Environment, Lund University, P.O. Box 118, SE-22100 Lund, Sweden; 2 Instituto de Planificación Urbana y Regional, Faculty of Architecture and Design, Catholic University of Santiago de Guayaquil, Av. Carlos Julio Arosemena Km. 1½ vía Daule, Guayaquil, Ecuador; 3 Aalto University, P.O. Box 11000, FI-00076 Aalto, Finland

**Keywords:** Outdoor thermal comfort, Microclimate, Subjective thermal comfort assessment, Warm-humid climate, Ecuador

## Abstract

The thermal environment outdoors affects human comfort and health. Mental and physical performance is reduced at high levels of air temperature being a problem especially in tropical climates. This paper deals with human comfort in the warm-humid city of Guayaquil, Ecuador. The main aim was to examine the influence of urban micrometeorological conditions on people’s subjective thermal perception and to compare it with two thermal comfort indices: the physiologically equivalent temperature (PET) and the standard effective temperature (SET*). The outdoor thermal comfort was assessed through micrometeorological measurements of air temperature, humidity, mean radiant temperature and wind speed together with a questionnaire survey consisting of 544 interviews conducted in five public places of the city during both the dry and rainy seasons. The neutral and preferred values as well as the upper comfort limits of PET and SET* were determined. For both indices, the neutral values and upper thermal comfort limits were lower during the rainy season, whereas the preferred values were higher during the rainy season. Regardless of season, the neutral values of PET and SET* are above the theoretical neutral value of each index. The results show that local people accept thermal conditions which are above acceptable comfort limits in temperate climates and that the subjective thermal perception varies within a wide range. It is clear, however, that the majority of the people in Guayaquil experience the outdoor thermal environment during daytime as too warm, and therefore, it is important to promote an urban design which creates shade and ventilation.

## Introduction

Global warming is especially problematic in tropical climates where people are subjected to longer periods of discomfort as well as an increased risk of experiencing health problems. This is an increasing problem in cities since much of the world’s urbanization is taking place in the tropics. Several studies in warm-humid climates have shown that the outdoor environment is very uncomfortable during daytime, especially between 11:00 and 16:00 (Johansson and Emmanuel 2006; Emmanuel et al. 2007; Johansson et al. 2013; Ndetto and Matzarakis 2013; Yahia et al. 2016).

Subjective outdoor thermal comfort has received increased attention in the latest decade, and the number of studies has increased each year (Johansson et al. 2014). Compared to indoors, the outdoor thermal environment is complex and there are large temporal and spatial variations; thermal conditions are spanning from thermal comfort to a thermally stressful environment (Spagnolo and de Dear 2003; Holst and Mayer 2011; Lee et al. 2014). Many studies have found that outdoors, the thermal comfort range is wider than indoors (e.g. Spagnolo and de Dear 2003; Lin 2009; Yahia and Johansson 2013). Thermal adaptation—which can include physiological, psychological and behavioural factors—has proven to play an important role in subjective thermal comfort assessment (Knez and Thorsson 2006; Lin 2009; Lin et al. 2011; Nikolopoulou 2011; Yahia and Johansson 2013).

There have been several studies recently on outdoor thermal comfort in urban areas in warm-humid climates. The major part of these studies has been in cities with warm-humid summers with temperate winters such as Chiayi and Taichung, Taiwan (Lin 2009; Lin et al. 2011), São Paulo, Brazil (Monteiro and Alucci 2012), Hong Kong (Ng and Cheng 2012) and Guangzhou, China (Xi et al. 2012). The study in Singapore (Yang et al. 2013) on the other hand treated an equatorial climate with warm-humid conditions all year round, but this study did not calculate any thermal comfort index. Many of the above-mentioned studies found evidence of thermal adaptation since the thermal comfort zone and/or neutral value of the chosen thermal index proved to be higher than for temperate climates (see also Johansson (2016)).

During the last century, well over 100 thermal indices have been developed (Blazejczyk et al. 2012; de Freitas and Grigorieva 2015). These indices were recently documented and classified by de Freitas and Grigorieva (2015). There is a distinct difference between the so-called rational indices, which are based on the heat balance of the human body, and simpler empirical and statistical indices. A comparison of thermal indices by Blazejczyk et al. (2012) showed that the rational indices standard effective temperature (SET*, when used outdoors sometimes referred to as OUT_SET*), the physiologically equivalent temperature (PET), the perceived temperature (PT) and the recently developed universal thermal climate index (UTCI) performed well and were highly correlated. On the other hand, some simpler empirical indices did not correlate very well with the more advanced rational indices.

PET is up to now the most commonly used index in outdoor thermal comfort studies (Johansson et al. 2014). Although the index is simpler than UTCI (de Freitas and Grigorieva 2016), it has proven to work well in warm-humid climates. One reason for this could be that the standard clothing and activity of the index are similar to what can be found in warm-humid climates.

SET* (or OUT_SET*) is also less advanced than UTCI. The index has the advantage that actual observed values of clothing insulation and metabolic rate can be used (de Freitas and Grigorieva 2016). SET* has been used in many outdoor thermal comfort studies (Johansson et al. 2014) including in warm-humid climates.

UTCI is the newest and the most advanced of the rational thermal indices. The index uses a multi-node model of human thermoregulation. The reduction of clothing insulation due to the wind speed is taken into account. The clothing value depends on the outdoor air temperature and is thus high at a low air temperature and low at a high air temperature. UTCI has a fixed value of activity (walking) and is thus aimed for outdoor studies (Blazejczyk et al. 2012).

This paper deals with microclimate and subjective human comfort in the warm-humid city of Guayaquil, Ecuador. The main aim is to examine the influence of urban micrometeorological conditions on people’s subjective thermal perception and to compare it with different thermal comfort indices. The aim is also to compare different types of public space in urban areas which are characterized by different microclimates and to compare the thermal perception between the dry and the rainy seasons.

This paper contributes to knowledge about the outdoor thermal environment of urban spaces in Guayaquil which have not yet been investigated from microclimate and thermal comfort perspectives. A novelty of this paper is that a distinct difference in thermal perception between the dry and wet seasons is found which indicates that other factors than thermophysiological ones influence thermal perception.

## Guayaquil and its climate

Guayaquil is the largest city of Ecuador with about 2.6 million inhabitants in the metropolitan area (Delgado 2013). The city is situated at sea level near the equator at latitude 2° 12′ S and longitude 79° 54′ W. Guayaquil’s historical centre was established during the Spanish colonial time to the west of the Guayas River, and its street pattern followed a regular orthogonal grid. Due to marshlands in the west and the river in the east, the city has grown mainly to the north and to the south, see Fig. 3.

**Fig. 1:**
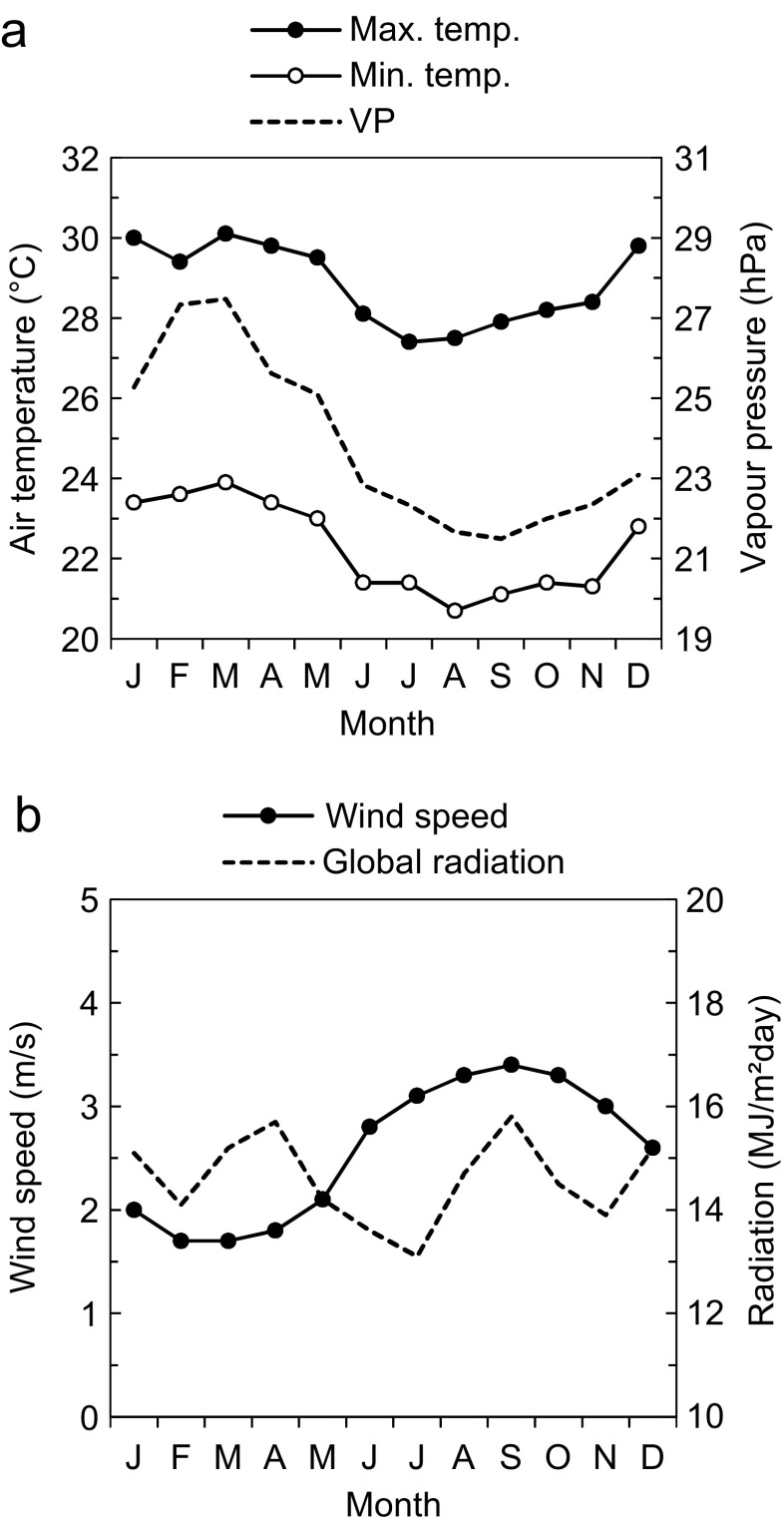
Climate of Guayaquil for the period 1964–1990. **a** Mean maximum and minimum air temperature and vapour pressure. **b** Mean daily wind speed and mean daily global solar radiation. Source: Meteotest (2014)

Being part of the coastal zone of Ecuador, Guayaquil has a warm-humid climate. Although the city is close to the equator, the climate is moderated by the cooling effect of the Humboldt Current along the west coast of South America. There are two distinct seasons: the rainy season from December to April and the dry season from May to November. Although precipitation is limited to 4–5 months, the humidity remains high all year round due to the proximity to the Pacific Ocean. The climate is very stable over the year with fairly high temperature and humidity of the air, see Fig. 1a. Wind speed is low, whereas solar radiation is fairly strong throughout the year, see Fig. 1b. The rainy season has the worst thermal conditions since both the air temperature and vapour pressure are higher and the wind speed is lower.

## Methodology

Micrometeorological measurements and questionnaire surveys were conducted simultaneously during the dry season (June 2009) and the rainy season (March–April 2010).

### Field survey locations

**Fig. 2:**
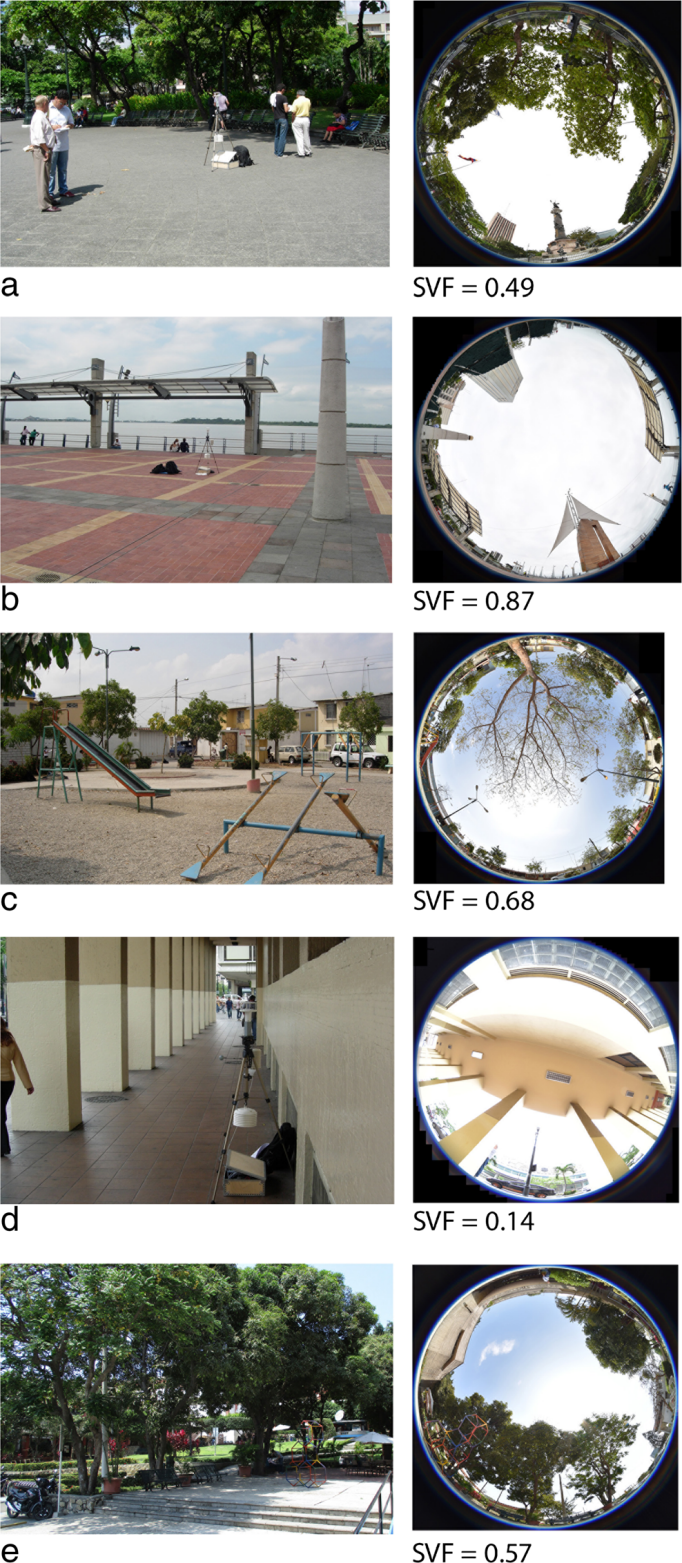
Photos and sky-view photos as well as corresponding sky-view factors (SVFs) for the locations of the field surveys. **a** Parque Centenário. **b** Malecón 2000 waterfront. **c** Neighbourhood square in Mucho Lote. **d** Arcade along Avenida 9 de Octubre. **e** Small square at the Catholic University of Guayaquil

**Fig. 3:**
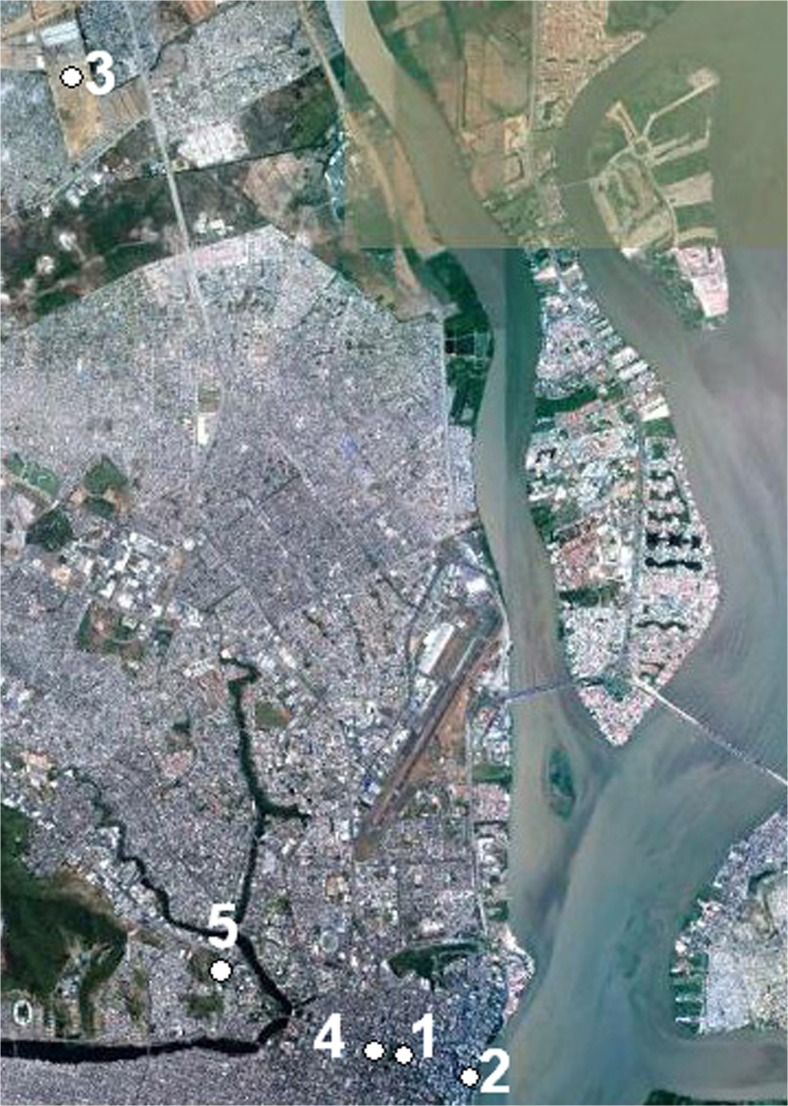
Google Earth picture of Guayaquil with sites marked. Parque Centenario (*1*), Malecón 2000 (*2*), Mucho Lote (*3*), Avenida 9 de Octubre (*4*), Catholic University of Guayaquil (*5*)

The field surveys were carried out in five public spaces of different urban design in Guayaquil: one park, one waterfront, two squares and one pedestrian arcade. The studied locations are shown in Fig. 2, and their geographical locations in Guayaquil are shown in Fig. 3.

Parque Centenario (site 1) is located in the historic centre of the city. The park has plentiful vegetation including large trees, and there are plenty of benches, many of which are situated in the shade under tree canopies. The measurement equipment was placed on a pedestrian path in the centre of the park (Fig. 2a).

Malecón 2000 (site 2) is a waterfront recreational area along the Guayas River. It is one of the most popular urban spaces in the city and runs along the eastern side of the historic centre. The measurement equipment was placed near the river (Fig. 2b).

Mucho Lote (site 3) is a recently constructed large urban development situated 12 km north of the city centre. The measurement equipment was placed at a small neighbourhood square with sparse vegetation (Fig. 2c).

Avenida 9 de Octubre (site 4) is an east–west-oriented avenue in the historic city centre who is aligned by pedestrian arcades, a traditional architectural element providing shaded pathways for pedestrians (Delgado 2013). The measurement equipment was placed on the pavement under an arcade (Fig. 2d).

The Catholic University of Guayaquil (site 5) is situated just west of the city centre. The measurement equipment was placed at a small square in the campus (Fig. 2e). (This site was only included in the rainy season.)

Fisheye photos of each site, taken at the spot where the measurement equipment was placed, are shown in Fig. 2. The most open site is the Malecón 2000 waterfront area with a sky-view factor (SVF) of 0.87, whereas the lowest SVF (0.14) is found for the pedestrian arcade.

### Micrometeorological measurements

During both the dry and rainy seasons, the field campaigns normally took place sometime between 11:00 and 16:00, i.e. during the hottest period of the day; however, a complimentary campaign was carried out during one evening in the dry season. Each campaign lasted for about 1 h. The dates and the times of each field survey are described in Table 1. The field surveys took place during clear, partly cloudy and overcast weather conditions. Table 2 shows the official meteorological conditions at the international airport for the measurement days.

**Table 1:** Measurement data for each field campaign including season, date, site, time, number of interviews as well as average values of air temperature, vapour pressure, mean radiant temperature and wind speed for the different measurement periods

Season	Date	Site no.	Description	Measurement period	No. of interviews	*T*_*a*,ave_ (°C)	VP_ave_ (hPa)	*v*_ave_ (m/s)	*T*_*g*,ave_ (°C)	*T*_*mrt*,ave_ (°C)
Dry	15 June 2009	1	Park	11:10–12:10	39	27.2	23.4	1.0	33.2	55.6
	16 June 2009	2	Waterfront	11:30–12:30	39	28.2	23.8	1.3	34.6	62.9
	18 June 2009	1	Park	11:50–13:05	45	25.8	22.6	0.7	28.5	37.3
	19 June 2009	3	Neighbourhood square	14:20–15:30	43	28.9	23.7	1.4	31.8	46.5
	24 June 2009	2	Waterfront	13:50–15:10	40	27.8	24.8	1.1	31.3	46.7
	25 June 2009	3	Neighbourhood square	13:30–14:30	43	30.9	22.6	1.1	36.3	58.3
	25 June 2009	2	Waterfront	18:30–19:40	48	27.0	25.7	1.6	26.8	25.1
	26 June 2009	4	Pedestrian arcade	13:30–14:30	46	27.1	22.7	0.6	27.9	30.4
Rainy	25 March 2010	5	Campus square	15:00–16:05	30	29.5	28.4	0.8	31.4	38.7
	31 March 2010	3	Neighbourhood square	14:10–15:20	27	30.6	29.5	0.9	35.2	52.2
	05 April 2010	1	Park	14:50–15:50	49	33.7	26.8	0.9	41.1	66.7
	06 April 2010	2	Waterfront	14:40–15:40	46	30.4	30.0	2.2	35.2	67.2
	07 April 2010	4	Pedestrian arcade	12:50–13:40	49	30.7	27.8	0.7	31.5	34.4

Air temperature (*T*_*a*_, °C), wind speed (*v*, m/s), globe temperature (*T*_*g*_), mean radiant temperature (*T*_*mrt*_). The vapour pressure (*VP*) was calculated based on *T*_*a*_ and relative humidity. (Geographical positions of the sites are shown in Fig. 2)

**Table 2:** Official meteorological data at the international airport for the measurement days

Season	Date	*T*_*a*,max_ (°C)	*T*_*a*,min_ (°C)	VP_ave_ (hPa)	Rainfall (mm)	*v*_ave_ (m/s)	*v*_max_ (m/s)
Dry	15 June 2009	31	22	22.7	0	3.3	6.7
	16 June 2009	31	23	22.3	0	2.9	5.7
	18 June 2009	27	22	21.2	0	3.5	7.7
	19 June 2009	29	20	22.0	0	3.1	6.2
	24 June 2009	29	22	20.8	0	2.7	4.6
	25 June 2009	30	21	21.1	0	3.6	6.7
	26 June 2009	27	21	21.5	0	3.1	5.7
Rainy	25 March 2010	32	25	26.6	1	2.2	4.1
	31 March 2010	31	24	27.9	13	1.0	2.6
	05 April 2010	33	23	26.0	9	1.8	4.1
	06 April 2010	34	25	28.2	29	2.2	5.1
	07 April 2010	32	26	27.7	0	2.7	5.7

Source: TuTiempo (2016)

Air temperature (*T*_*a*_, °C), wind speed (*v*, m/s). The vapour pressure (VP) was calculated based on average *T*_*a*_ and average relative humidity

All four micrometeorological variables which influence thermal comfort were obtained from a mobile measurement station. Air temperature (*T*_a_), relative humidity (RH) and wind speed (*v*) were measured directly, whereas the mean radiant temperature (*T*_mrt_) was calculated from measured values of globe temperature (*T*_g_), *T*_a_ and *v*. *T*_mrt_ considers both short-wave and long-wave radiation and represents the weighted average temperature of an imaginary enclosure that gives the same radiation as the complex urban environment (ISO 7726 1998).

**Fig. 4:**
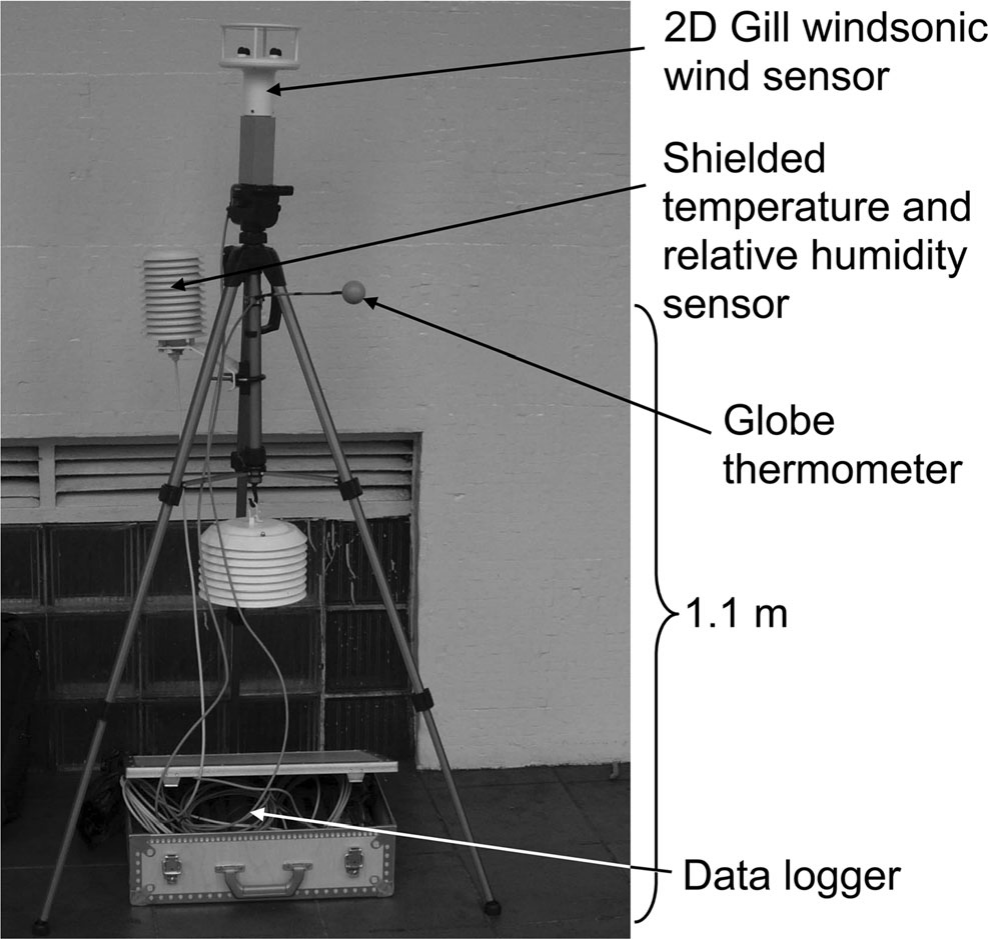
The measurement equipment used in the field campaigns (Johansson and Yahia 2011)

The sensors were connected to a data logger (Campbell CR800) with which 1-min averages were sampled. The sensors were mounted on a camera tripod as shown in Fig. 4. The measurements were taken at the height of 1.1 m, except for the wind speed which was measured at 1.5-m height.

The wind speed was measured with a two-dimensional ultrasonic sensor (Gill windsonic) which detects speeds down to 0.02 m/s. However, it measures only the horizontal component of the wind speed, and since the urban wind pattern is very irregular and includes vertical movements, it is likely that the wind speed was slightly underestimated. The corresponding wind speed at 1.1 m was calculated as (Aynsley et al. 1977)

1 $$ {v}_{1.1}={v}_{1.5}\times {\left(\frac{1.1\ \mathrm{m}}{1.5\ \mathrm{m}}\right)}^{\propto } $$

where *v*_1.1_ is the wind speed at 1.1 m, *v*_1.5_ is the wind speed at 1.5 m and *α* is the mean speed exponent which depends on the roughness of the ground (e.g. *α* = 0.25 for parks and suburbs and *α* = 0.36 in the centre of large cities (Aynsley et al. 1977)). This formula is valid for an open terrain and may be less accurate in a complex urban context. Nevertheless, we believe that the wind speed is fairly accurate since the difference in wind speed between 1.1 and 1.5 m is small.

The air temperature and humidity sensor (Rotronic Hydroclip S3) had a rather long response time and was therefore let 5 min in the actual ambiance before measurements started. The sensor was covered by a white, naturally ventilated radiation shield.

The globe thermometer consisted of a quick responding pt100 sensor inside a plastic (celluloid) table tennis ball painted flat grey (colour RAL 7001) according to Thorsson et al. (2007b). Its diameter, *D*, was 40 mm; its weight about 3 g, and its emissivity, *ε*, was assumed to be 0.97. Thorsson et al. (2007b) calibrated the thermometer in a study in Göteborg, Sweden, with measurements of short-wave and long-wave radiation in six directions (downward, upward, north, east, south and west) which yielded the following formula to calculate *T*_mrt_:

2 $$ {T}_{mrt}={\left[{\left({T}_g+273.15\right)}^4+\frac{1.335\times {10}^{8\ }{v_a}^{0.71}}{\varepsilon {D}^{0.4}}\times \left({T}_g-{T}_a\right)\right]}^{0.25}-273.15 $$

where *T*_*g*_ = globe temperature (°C), *v*_*a*_ = wind speed (m/s), *T*_*a*_ = air temperature (°C), *D* = globe diameter (m) and *ε* = globe emissivity.

Due to its low weight, this globe thermometer has a time constant of about 5 min (Nikolopoulou et al. 1999) which is much shorter than for the standard indoor 150-mm cupper globe thermometer which has a time constant of 20–30 min (ISO 7726 1998; McIntyre 1980). It should be noted that the *T*_*mrt*_ calculated in this way is very sensitive to variations in wind speed, e.g. an increase in wind speed will make the globe cool down and *T*_*g*_ decrease, but as this will take several minutes, *T*_*mrt*_ will be overestimated. Similarly, a sudden decrease in wind speed will lead to an underestimated *T*_*mrt*_. To reduce the sensitivity to wind speed variations, 10-min averages of wind speed and 5-min averages of air and globe temperature were used in the calculations of the *T*_*mrt*_. The air and globe temperature probes were calibrated in climate chambers to determine any systematic differences between them. A difference of about 0.4–0.5 °C was found, and this was corrected for in Eq. (2).

### Thermal comfort investigation

**Fig. 5:**
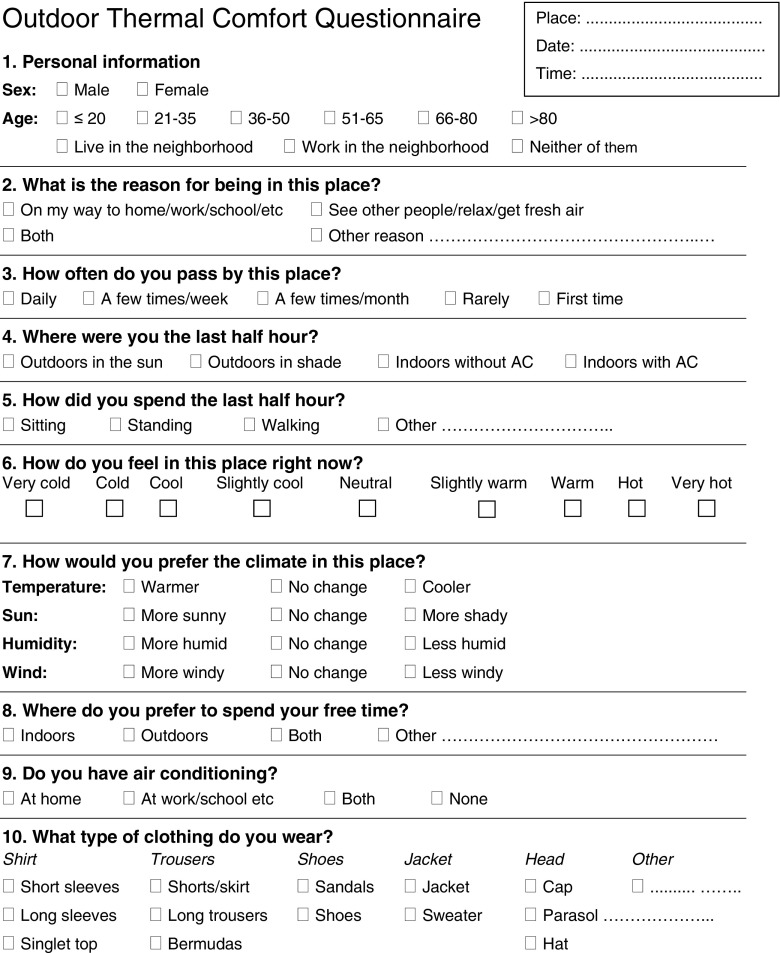
The English version of the questionnaire used in the field campaigns

The questionnaire surveys to estimate the subjective thermal comfort were performed simultaneously with the measurements at each location. The English version of the questionnaire is shown in Fig. 5; during the field campaigns, a Spanish version was used. The subjective thermal perception of the subjects was reported on a nine-point scale. The reason for using a nine-point scale instead of the commonly used seven-point scale was that the conditions outdoors are more extreme than indoors. This is in accordance with ISO 10551 (1995). The two scales are in fact identical except that the nine-point scale has added very cold (−4) and very hot (+4), thus two extreme values. A nine-point scale was also used by Thorsson et al. (2007a), Kántor et al. (2012), Yahia and Johansson (2013) and Cohen et al. (2013). Due to the warm climate in Guayaquil, there were virtually no votes below “slightly cool (−1)”, so in reality, only six points were used. It was emphasized by the interviewers that it was the subjects’ perception at the moment of the interview that was requested and not their general opinion.

People’s preference to different weather variables was reported on a three-point scale according to McIntyre (1980), see Fig. 5.

Apart from thermal perception and preference, the questionnaire included questions about gender, age, reason for being in the place, time spent outdoors as well as whether the subjects had air conditioning at home and in their office/school, see Fig. 5. The type of clothing and activity were observed by the interviewer and chosen from a predefined list. The total clo value for the clothing ensemble was then calculated according to ISO 7730 (2005). The activity before the interview was defined as sitting, standing, walking slowly, etc. However, during the interview, the subjects were normally standing. The actual activity was therefore taken as an average between the previous activity and standing corresponding to activity levels of 1.1 met for sitting/standing, 1.2 met for standing/standing and 1.5 met for walking slowly/standing.

The team carrying out the interviews consisted of the main author and a team of staff members and students from the Faculty of Architecture and Design at the Catholic University of Guayaquil. One interviewer normally participated in two to four surveys. The team thus shifted a bit in composition from site to site but always consisted of a mixture of men and women. At each site, the interview team consisted of three to six persons. The interviewers were asked to interview any person on the street regardless of age and gender. The number of interviewed persons at each site varied between 27 and 49, see Table 1.

At all sites, except under the arcade on Avenida 9 de Octubre, the measurement equipment was fully exposed to solar radiation. The subjects were interviewed as close to the measurement equipment as possible. This means that the subjects were exposed to solar radiation (if the sky was not overcast) at all sites except Avenida 9 de Octubre. In a few cases though, people sitting on the benches nearby, mainly in shade, were also interviewed.

From the total of 596 conducted questionnaires, several were excluded. This included foreign tourists as well as Ecuadorians from the much colder mountain region (*la Sierra*). People from other parts of the coastal region, even if not living in Guayaquil, were however included. Some strange/inconsistent questionnaires were also excluded from the sample.

This study presents the results from 343 questionnaires answered during the dry season and 201 during the rainy season. The two samples were similar in terms of gender and age distribution. About 36% of the respondents were women. The interviewed subjects were young or middle age in general; the largest age group was 21–35 years (36%) followed by 36–50 years (26%).

### Thermal comfort assessment

Thermal comfort was assessed using the PET and the SET*. The reason for choosing these indices is that both are based on the heat balance equation of the human body and have been commonly used in outdoor thermal comfort studies (Johansson et al. 2014). This makes it easier to compare the results of this study with previous studies. PET, which takes air temperature, humidity wind speed and *T*_*mrt*_ into account, is defined as the air temperature at which the energy balance for typical indoor conditions is balanced with the same mean skin temperature and sweat rate as calculated for the complex outdoor conditions (Mayer and Höppe 1987). SET*, which apart from the four meteorological variables which affect thermal comfort also takes clothing and activity into account, represents the air temperature of a standard environment with 50% relative humidity for subjects having standardized clothing for the given activity in the real environment. In this standard environment, the skin temperature, skin wettedness and heat loss are the same as in the real environment (Gagge et al. 1986). Table 3 shows the suggested thermal perception scales of PET and SET*.

**Table 3:** Suggested thermal perception scales of PET and SET* (Blazejczyk et al. 2012; Matzarakis et al. 1999; McIntyre 1980)

PET (°C)	SET* (°C)	Thermal perception	Stage of stress
<4	<10.0	Very cold	Extreme stress
4–8	10.0–14.5	Cold	Strong stress
8–13	14.5–17.5	Cool	Moderate stress
13–18	17.5–22.2	Slightly cool	Slight stress
18–23	22.2–25.6	Comfortable	No stress
23–29	25.6–30.0	Slightly warm	Slight stress
29–35	30.0–34.5	Warm	Moderate stress
35–41	34.5–37.5	Hot	Strong stress
>41	>37.5	Very hot	Extreme stress

It should be noted that the PET scale is not based on field surveys; it is a transformation of the predicted mean vote scale for an internal heat production of 80 W and a clothing insulation of 0.9 clo (Matzarakis et al. 1999)

Although being the most advanced index and having a very good physiological basis, UTCI was not used in the present study. The reason is that the index, in its present version, has a number of weaknesses, e.g. the fixed level of activity (walking at a speed of 1.1 m/s corresponding to 2.3 met) (Blazejczyk et al. 2012) is higher than what can be expected in a warm-humid climate, and the clothing values are unrealistically low at high levels of air temperature as shown by Pantavou et al. (2013). Moreover, the wind speed used in the calculation is the value at 10 m a.g.l. and not at pedestrian height (Blazejczyk et al. 2012).

#### Thermal comfort zones

Normally, votes outside the three central categories (−1, 0 and +1) of the thermal perception scale are considered to be unacceptable votes (e.g. Spagnolo and de Dear 2003; Lin 2009). In this study, the comfort limits for 20% unacceptability were calculated.

#### Neutral index value

The index value found by statistical analysis to most frequently coincide with the thermal perception vote “0” (neutral or comfortable) is referred to as the sample’s neutral index value (Spagnolo and de Dear 2003; Lin 2009). To determine the neutral index value, probit technique (Ballantyne et al. 1977) was used. The neutral value of each index was determined by grouping all thermal perception votes <0 into a group of cooler than neutral and all votes >0 into a group of warmer than neutral. The votes equal to 0 were split randomly between the two groups. The neutral index value was then determined as the value at which 50% of the sample voted cooler than neutral and 50% voted warmer than neutral. Statistical Package for the Social Sciences (SPSS) was used to perform the probit analysis.

#### Preferred index value

The preferred index value was determined as the value at which 50% of the sample voted that they wanted it to be cooler and 50% voted that they wanted it to be warmer. It was calculated in the same way as the neutral index value above.

## Results and discussion

### Seasonal and intra-urban microclimatic differences

A proper comparison of the micrometeorological conditions between the different areas was hampered by the fact that the field campaigns were carried out on different days for each site and at different times of the day. However, as Table 2 shows, the official meteorological conditions of Guayaquil were fairly stable during the measurement days.

Both the official meteorological conditions (Table 2) and the measured micrometeorological conditions (Table 1) show a clear difference between the dry and rainy seasons. Both air temperature and vapour pressure are higher during the rainy season, whereas the wind speed is lower.

Some distinctive differences between the sites were found. The vapour pressure was higher at the Malecón 2000 site near the river. Open sites without obstructions in the form of large buildings such as Malecón 2000 and Mucho Lote were windier than the centrally located sites. *T*_*mrt*_ also varied considerably between the sites, which was partly due to the sky conditions. During clear sky conditions, *T*_*mrt*_ could vary between 60 and 70 °C, whereas during cloudy conditions, *T*_*mrt*_ varied between 35 and 40 °C. The lowest *T*_*mrt*_ was found under the pedestrian arcade in Avenida 9 de Octubre being only around 30 °C. The decisive effect of shading on *T*_*mrt*_ and consequently thermal perception has been shown in several other studies, e.g. Johansson et al. (2013), Lee et al. (2013), Lee et al. (2016) and Yahia et al. (2016).

### Subjective thermal comfort

**Fig. 6:**
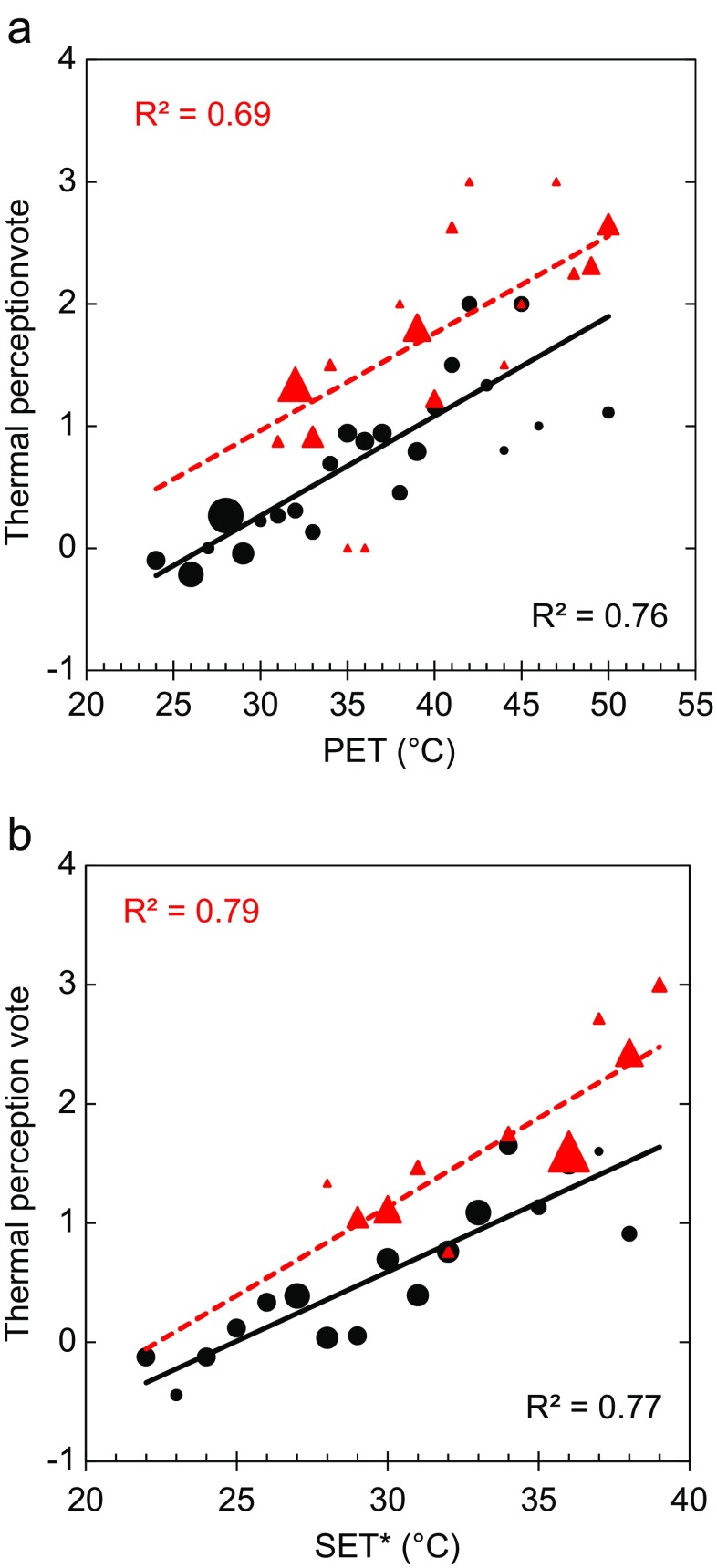
Relationship between **a** PET and **b** SET* and the thermal perception votes for the dry season (*black markers*, *n* = 343) and the rainy season (*red markers*, *n* = 201). The size of the markers is proportional to the number of votes in each bin. Very hot (*4*), hot (*3*), warm (*2*), slightly warm (*1*), comfortable (*0*), slightly cool (−1)

Figure 6 shows the relationship between thermal perception votes and PET and SET* for the dry and rainy seasons. As expected, there is a tendency of increased thermal stress with increasing PET and SET*. Similar relationships have been found by, e.g. Cohen et al. (2013), Kántor et al. (2012), Lin (2009), Lin et al. (2011), Ng and Cheng (2012) and Yahia and Johansson (2013). In general, both seasons follow the same pattern, but a distinct difference in thermal perception between the dry and rainy seasons can be seen, both for PET and SET*. This difference may have several reasons. It may be that in the end of the rainy season, which is very uncomfortable, people are tired after a long period of discomfort and therefore vote as if it was more uncomfortable than it actually is. It might also be that although the questioner emphasized that the opinion asked for was at the very moment of the interview, some people may have voted according to how they perceive the environment rather than their personal perception as discussed by Spagnolo and de Dear (2003).

As shown in Fig. 6, SET* has a slightly better correlation than PET, perhaps due to the fact that clothing and activity are included in SET*. A better correlation for SET* compared to PET was found by Yahia and Johansson (2013) as well. The reason why PET has almost as good correlation as SET* is probably that the effect of differences in clothing and activity is small in the case of Guayaquil. People tend to dress similarly, and the activity did not vary much between the subjects.

**Fig. 7:**
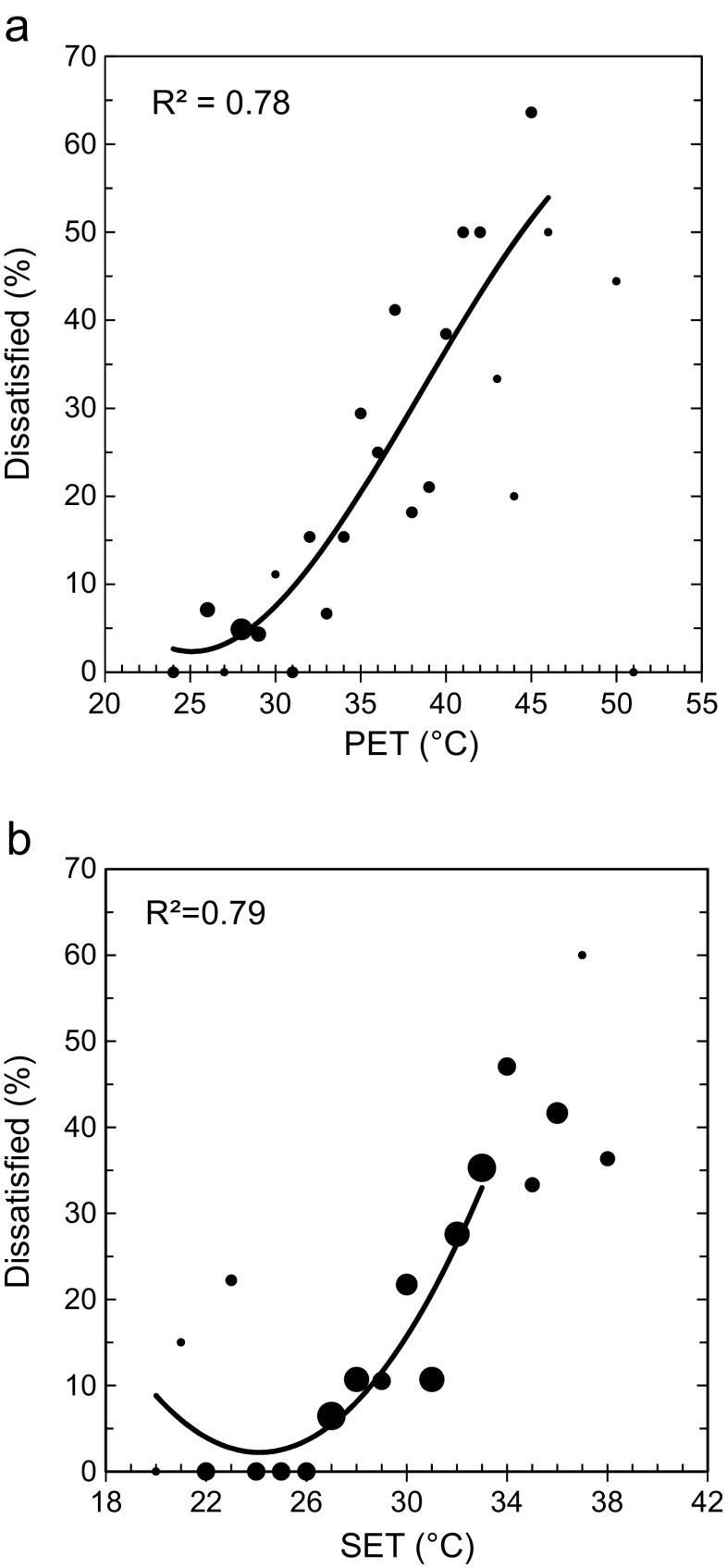
Relationship between the percentage of dissatisfied and **a** PET and **b** SET* during the dry season. The size of the markers is proportional to the number of votes in each bin

Figure 7 shows the relationship between PET and SET* and the percentage of people being dissatisfied, that is outside the central votes. Since this investigation took place in a warm climate and since the field surveys were mainly concentrated to the hottest period of the day, the curves in Fig. 7 are dominated by index values warmer than “comfortable”. Thus, only the upper limits of acceptability (comfort) were possible to determine for each index.

Table 4 shows the upper comfort limits for 20% unacceptability (80% acceptability) and the neutral and preferred values for SET* and PET during the dry season and the rainy season as well as for both seasons combined.

**Table 4:** Upper comfort limits (20% unacceptability) and neutral and preferred index values during the dry season and the rainy season as well as for both seasons combined

	Upper comfort limit			Neutral index value			Preferred index value		
	Dry season	Rainy season	Both seasons	Dry season	Rainy season	Both seasons	Dry season	Rainy season	Both seasons
PET	34.3	25.3	31.3	26.9	*21.9*	25.7	*15.5*	*18.6*	*17.5*
SET*	30.9	25.3	29.0	24.5	*20.5*	24.3	*17.7*	*23.3*	18.6

Values in italics are not significant at 95% confidence

It is clear from Table 4 that people tend to accept much higher values of PET and SET* than the “original” thermal perception scale of the index (see Table 3).

Thermal comfort is a concept that certainly many of the respondents were not familiar with. It is indeed a complex concept as not only air temperature but many other environmental, personal and psychological factors are involved. One should also bear in mind that the climate of Guayaquil is very stable, the variation from 1 day to another is small, which means that weather and comfort are not “an issue”. It seems, however, that the great majority of the respondents have understood the key question: How do you feel at this moment in this place?

**Fig. 8:**
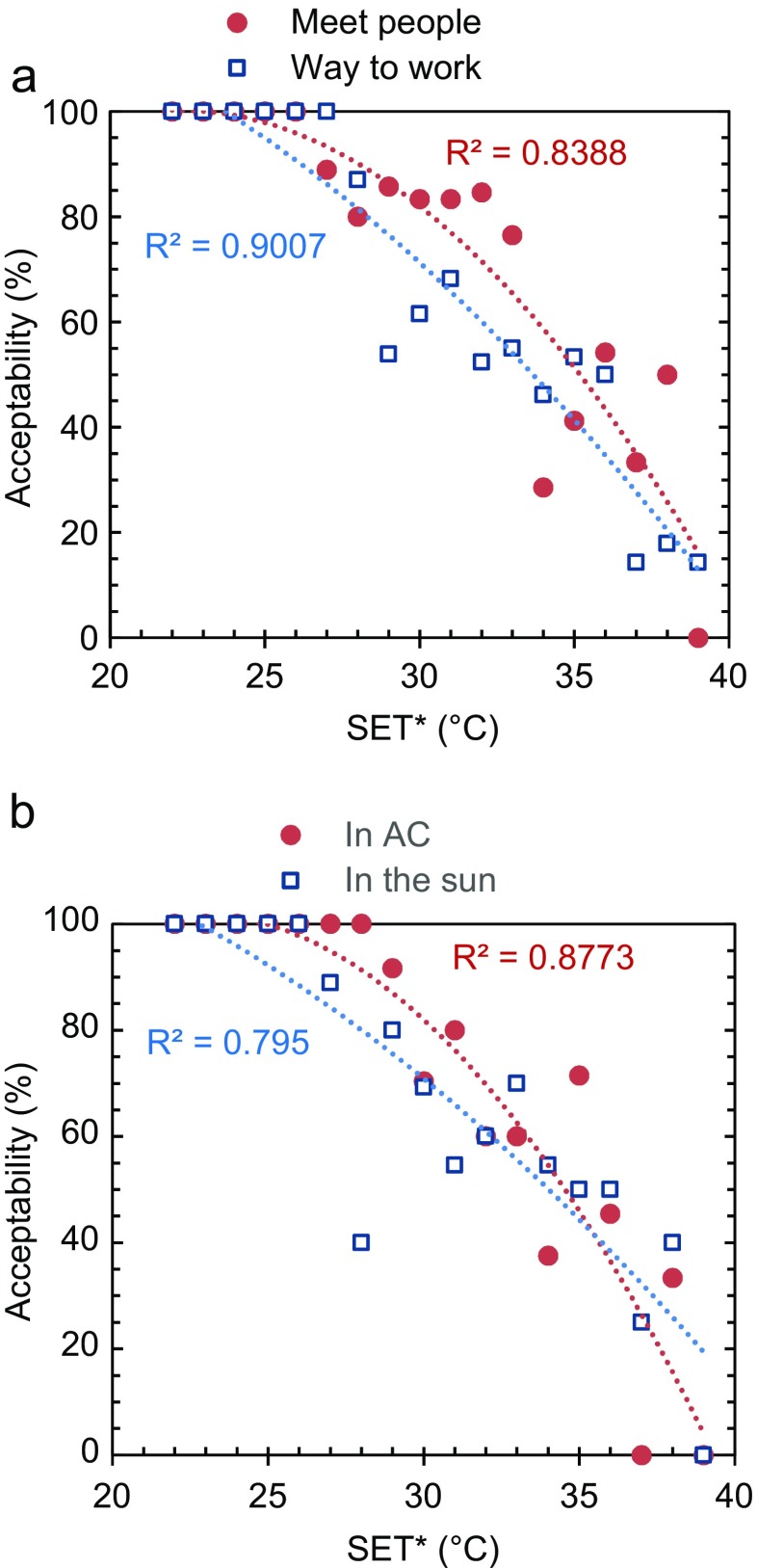
Relationship between the level of acceptability and SET* for **a** subjects who went to the place by their free will to meet other people versus people who passed by the place on their way to work, school, etc. and **b** subjects who spent the time before the interview indoors in air-conditioned space versus those who were outdoors exposed to the sun. (Average of both dry and rainy seasons)

The reason for being in the place where the interview took place turned out to have a significant impact on the subjects’ thermal perception. Figure 8a shows the relationship between the level of acceptability and SET* for people who went to the place by their free will to meet other people (37% of the sample) and people who passed by the place on their way to work, school, etc. (52% of the sample). The people being in the place voluntarily had a much higher acceptance of high SET*; at the level of 80% acceptability, the subjects who came to meet people accepted a SET* of about 31 °C, whereas people who had to be there accepted only about 28 °C.

### Influence of air conditioning on thermal perception

The thermal condition of the environment where the subjects have been before the interview may influence their thermal perception. Figure 8b shows a comparison between subjects who had been indoors in air-conditioned space just prior to the interview (30% of the sample) and those who had been the last half hour, or longer, in the sun (22% of the sample). At the level of 80% acceptability, the subjects who came from an air-conditioned space accepted higher SET* values (about 30.5 °C) than those who had been exposed to the sun (about 28 °C). Possibly, this is because the skin and body core temperature of the subjects coming from the air-conditioned space were considerably lower than for those who had stayed in the hot outdoors for a longer time. The outdoor subjects may have been closer to physiological discomfort thresholds. The result agrees with Ng and Cheng (2012) who also found that subjects having been exposed to air conditioning prior to the questionnaire survey were more tolerant to heat stress. The effect of the influence of thermal history found in this study may also explain why some people voted “very hot” although the measurements showed only moderately warm.

It has been shown that people who spend a lot of time in air-conditioned spaces, e.g. having air conditioning both at home and at work, tend to be more sensitive to a high indoor air temperature (see, for example, Busch 1992). Yahia and Johansson (2013) found a similar tendency in the urban outdoors in Damascus. In this study, 61% had air conditioning at home or at work or both, whereas 39% did not have air conditioning at all. However, no clear difference could be found as regards the acceptance of high values of PET or SET* between these two groups.

### Preferred conditions

**Fig. 9:**
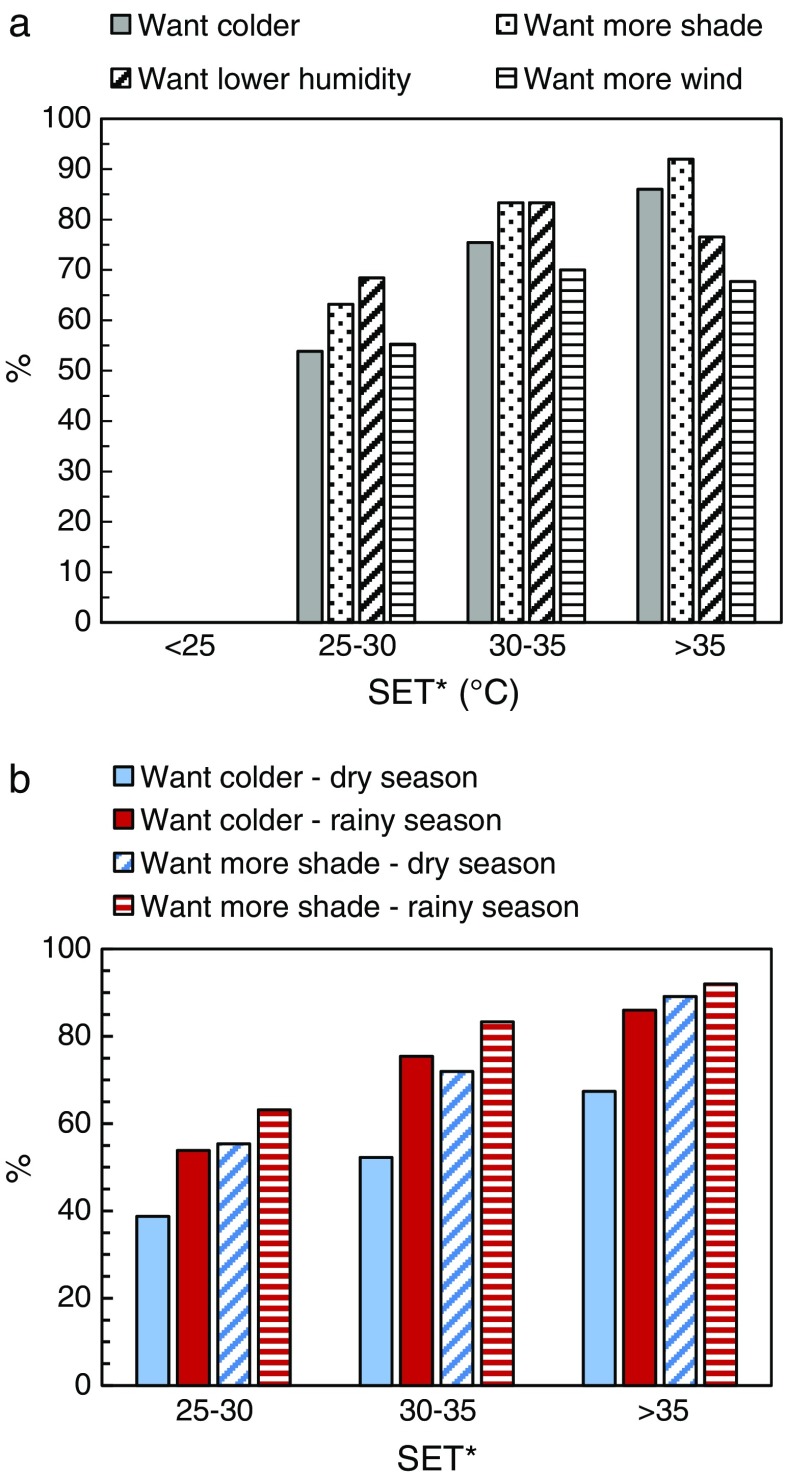
Relationship between SET* and preference. **a** The preference of temperature, humidity, shade and wind (average of both dry and rainy seasons). **b** The preference of temperature and shade for the dry and rainy seasons

Figure 9 shows the relationship between SET* and preference; it is clear that most people want it to be cooler and they want more shade, less humidity and more wind (Fig. 9a). As expected, the demand for lower air temperature, more shade and more wind in general increases with increased SET*, especially above 30 °C. This tendency is more pronounced in the rainy season as can be seen in Fig. 9b.

In general, the preferred index value was much lower than the neutral index value (Table 4). This agrees well with other studies in warm-humid climates (e.g. Lin 2009, 2011; Yang et al. 2013). This phenomenon, which is a result of people in a hot environment wanting it to be cooler, is often referred to as alliesthesia (Spagnolo and de Dear 2003). It may also be a result of people wanting stimulation (Nikolopoulou 2011), in this case a change from the constantly warm conditions to a cooler environment.

### Clothing

The clothing insulation varied between 0.2 and 0.9 clo for the whole sample, but about 80% of the observed clo values were between 0.4 and 0.6. The typical clothing ensemble for men was short-sleeved shirt, long trousers and shoes, which corresponds to a clo value of about 0.49. Even long-sleeved shirts were quite common. The typical female clothing was short-sleeved shirt, skirt or long trousers and sandals corresponding to 0.47 clo. Some people, most of them men, used a formal suit (corresponding to clo = 0.9). There was a slight difference in clothing between the dry and the rainy seasons: clo values were 0.02 and 0.06 lower for men and women respectively during the rainy season.

Although the amount of clothing was slightly lower than found during the warm-humid summer in Taiwan (around 0.6 clo, Lin 2009), it was higher than found in the summer in Lisbon (clo = 0.4, Andrade et al. 2011) and in Singapore (clo = 0.3, Yang et al. 2013). The reasons for the relatively heavy clothing might be that there is a special dress code at work, at school, etc. Another reason might be solar protection; some people actually claimed they used long-sleeved shirt to protect the skin against the sun. In the recreational area Malecón 2000, the clothing was slightly lighter than in the city centre with a higher presence of shorts, sleeveless shirts and sandals also for men.

Some men use the traditional *guayabera*. This is a shirt of light weight fabric, normally white in colour, which is worn untucked. It is well adapted to the climate as it reflects solar radiation well and allows convective heat loss. However, it is not so much used any more. Broad-brimmed hats were also more common in former times.

### Thermal adaptation

The fact that people accept PET and SET* values up to 31 and 29 °C respectively (Table 4) shows that the people of Guayaquil are thermally adapted to the local climate.

Some behavioural adaptation was also observed, e.g. during strong sunshine, people in public places was seeking shade, either under trees or man-made shading devices. Similar adaptive behaviour was found by Lin (2009) in Taiwan. Attributes to reduce solar radiation, especially in the face, were not used by the majority. However, some people used a cap, and some women used umbrella/parasol to protect themselves against the sun. One could also see people protecting their head by holding a notebook or briefcase against the sun.

## Conclusions

This study examined the influence of urban microclimate on people’s subjective thermal perception in the warm-humid city of Guayaquil. The two thermal comfort indices tested, the PET and the SET*, behaved similarly with a fairly good correlation to subjective votes of thermal perception.

The micrometeorological conditions varied between the five studied sites especially as regards the wind; the riverside area and the suburban low-rise neighbourhood had a higher wind speed than the centrally located sites. The most important feature of public spaces is however the availability of shade due to the intense solar radiation.

The people perceive the climate as uncomfortably warm, especially during the rainy season. For both indices, the neutral values and upper thermal comfort limits were lower during the rainy season. This seasonal difference in thermal perception suggests that not only thermal conditions but also psychological factors influence the thermal perception.

In order to use the PET and SET* as indicators of thermal comfort, the ranges for different states of thermal perception such as comfortable, “slightly warm”, etc. of each index need to be adjusted to the local climate. This study has defined the upper limits of the thermal comfort zones of PET and SET* and should be seen as a first attempt to define the outdoor thermal comfort zone for Guayaquil.

It was found that both the reason to be in a place as well as thermal history influenced the thermal perception. Subjects that visited a place voluntarily to meet other people accepted higher PET and SET* values than subjects who were passing by on their way to work, etc. Subjects that had been indoors in air-conditioned space recently before entering the urban space accepted higher PET and SET* values than those who had been outdoors for some time exposed to the sun.

The study showed that clothing seemed to be virtually independent of the weather conditions. It seemed to be more linked to the social context, whether being at work or school or performing some kind of leisure activity.

Finally, it is concluded that it is important to promote an urban design which creates shade and ventilation. This includes the use of elements such as shading devices, e.g. arcades and shading trees as well as to consider the prevailing wind direction.
